# Cognitive-behavioural therapy for the management of inflammatory bowel disease-fatigue: a feasibility randomised controlled trial

**DOI:** 10.1186/s40814-019-0538-y

**Published:** 2019-12-10

**Authors:** Micol Artom, Wladyslawa Czuber-Dochan, Jackie Sturt, Hannah Proudfoot, Danniella Roberts, Christine Norton

**Affiliations:** 1 Florence Nightingale Faculty of Nursing, Midwifery and Palliative Care, King’s College London, James Clerk Maxwell Building, 57 Waterloo Road, London, SE1 8WA UK; 2 Tobacco & Alcohol Research Group, University College London, London, UK

## Abstract

**Background:**

Fatigue is the third most prevalent symptom for patients with inflammatory bowel disease (IBD), yet optimal strategies for its management are unclear. Treatment protocols for fatigue in other conditions have been based on cognitive-behavioural models. Targeting cognitions, emotions and behaviour related to fatigue through cognitive-behavioural therapy (CBT) may be a viable option to improve fatigue and quality of life (QoL) in IBD.

**Methods:**

This single centre, two-arm, feasibility randomised controlled trial (RCT) aimed to assess the feasibility and initial estimates of potential efficacy of a CBT intervention for the management of IBD-fatigue. Feasibility, acceptability and initial estimates of potential efficacy outcomes were collected through self-report measures and semi-structured interviews. Participants were recruited from one tertiary referral centre. Intervention Group 1 received a CBT manual for fatigue, one 60-min and seven 30-min telephone sessions with a therapist over 8-weeks. Control Group 2 received a fatigue information sheet without therapist support. A nested qualitative study evaluated patients’ and therapists’ experiences, and IBD-healthcare professionals’ (HCPs) perceptions of the intervention.

**Results:**

Eighty-nine participants were assessed for eligibility. Of these, 31 of the 70 eligible participants consented to participate (recruitment rate of 44%). Of the 15 participants randomised to the intervention group, 13 (87%) started it and 10 (77% of those who started) completed all 8 sessions. Follow-up questionnaires were completed by 22 (71%) participants at 3 months, 14 (45%) at 6 months and 12 (39%) at 12 months’ follow-up. The intervention was acceptable to participants and feasible for therapists to deliver. HCPs reported that the intervention would be applicable, but time, finance and training constraints limit its implementation. Initial estimates of potential efficacy with complete case analysis showed a reduction in fatigue and an increase in QoL at 3, 6 and 12 months post-randomisation.

**Conclusions:**

A full-scale effectiveness RCT testing CBT for IBD-fatigue is feasible and is potentially worthwhile with some changes to the protocol. However, given the small numbers, further pilot work is warranted before a full-scale RCT.

**Trial registration:**

Registration Trial ISRCTN 17917944, Registered 2 September 2016

## Background

Inflammatory bowel disease (IBD) is a group of chronic, inflammatory conditions of the gastrointestinal tract. The two main types are Crohn’s disease (CD) and ulcerative colitis (UC) [1]. The clinical course of IBD is marked by exacerbation and remission [2, 3]. Its cardinal symptoms include diarrhoea, abdominal pain, urgency, tenesmus, weight loss and fatigue. CD may also lead to intestinal obstruction due to fistulae, strictures or abscesses [4, 5]. IBD affects about 300,000 people in the United Kingdom (UK) [6] and 2.2 million people in Europe [7]. IBD can have a negative impact on quality of life (QoL), with adverse effects on work, relationships and education [8]. In line with the international expert consensus of the Therapeutic Targets in Inflammatory Bowel Disease (STRIDE) initiative [9] on treatment targets for IBD, having a more holistic approach to its management which addresses patient reported outcomes [10] and recognises its psychological burden is thus an important aspect of care [11].

Fatigue is the third most predominant concern for patients with IBD [12], experienced by 44–86% of patients with active disease and 22–41% of patients in remission [13]. As patients struggle with fatigue in between flare-ups [14], patients in remission should not be overlooked [15]. Fatigue has been defined as a ‘persistent overwhelming sense of tiredness, weakness or exhaustion’ [16] that can be mental, physical or both [17]. It can have a negative impact on personal and social life, on work and employment and the ability to think clearly [18–20]. Fatigue and the development of fatigue management interventions are currently top IBD research priorities in the UK [21] and in Europe [22]. However, fatigue is only identified and managed in a small proportion of those affected [23]. The aetiology of fatigue is not well understood [13, 24]. Inflammation [25], disease activity [17, 26, 27] and anaemia [25, 28] can be predictive of fatigue. IBD-fatigue has also been linked to psychosocial factors including: depression and anxiety [17, 29, 30]; negative perceptions, cognitions and behaviours [31]; and sleep problems [29, 32, 33]. In a significant proportion of people there is no physiological explanation for their fatigue [34] and the ways in which clinical and psychosocial factors interact with each other to cause fatigue have rarely been explored [31].

Optimal strategies for the management of IBD-fatigue are unknown and fatigue has seldom been the primary outcome of trials [35–38] . Some benefits have been shown by pharmacological interventions utilising biologics [39–41], thiamine [38] and ferumoxytol [42]. However, it is unclear whether these results were due to a direct effect on fatigue or a reduction in inflammation in patients with active disease [43]. The only trial [37] examining the effect of physical activity, provided inconclusive results. Psychosocial interventions, including stress-management [44], solution-focused therapy [35, 36] and brief behavioural therapy for sleep [45] have shown promising effects but these declined over time. As there are similarities between the perceived experiences of fatigue in different long-term conditions [13, 46, 47], integrating current best evidence across conditions can help to identify effective interventions for IBD-fatigue and their underlying mechanisms [48] without ‘reinventing the wheel’ [49]. The majority of psychological treatment protocols in other conditions have been based on cognitive-behavioural models [50, 51], according to which symptoms are maintained by maladaptive cognitive and behavioural factors [52]. Disease-related or clinical factors trigger fatigue; the ways in which people respond cognitively, emotionally and behaviourally to their fatigue may then contribute to the perpetuation or worsening of symptoms [50]. The targeting of cognitions, emotions and behaviour related to fatigue through cognitive-behavioural therapy (CBT) may therefore be a viable option to improve clinical and psychosocial outcomes [53].

A recent systematic review and meta-analysis appraised psychological interventions for people with IBD [54] with encouraging short-term results for CBT on QoL and depression but no effects on IBD disease activity. Although CBT has not been used for fatigue, results from our previous study [31] show that the ways patients perceive, interpret and react to fatigue symptoms in IBD are largely comparable to patients with other conditions such as multiple sclerosis (MS) [55]. IBD-patients who have more negative perceptions of fatigue and higher levels of maladaptive behaviours have significantly greater fatigue levels [31]. Furthermore in a recent qualitative study, people with IBD described an all or nothing behavioural response, where they felt fatigued because they took on more whilst feeling well to compensate in advance for future periods of reduced functioning [56]. A CBT manual developed by Van Kessel et al. [57] for MS was therefore used as the foundation of the intervention for the current study. It was chosen for its promising results in reducing fatigue with MS patients [57, 58], its strong theoretical grounding in a CBT model [59] and its valuable in-depth mediation analysis of processes of change [60]. In the original study, 72 patients with MS-fatigue were randomised to 8 weekly, 50-min face-to-face sessions of CBT or relaxation training (RT). The CBT group reported significantly greater reductions in fatigue 9 months post-intervention compared to the RT group, with calculated effect sizes from baseline to the end of treatment of 3.03 for the CBT group and 1.83 for the RT group.

In the current study, the UK Medical Research Council (MRC) framework for the development of complex interventions [61] was utilised to guide the development of the intervention. Its content was based on cognitive-behavioural theory and was grounded in empirical evidence from our previous systematic reviews [47, 62], qualitative [13, 24, 46] and quantitative studies in IBD-fatigue [31]; and studies on MS-fatigue [57, 58]. Iterative work with people with IBD and healthcare professionals (HCPs) took place to tailor the intervention to their needs. A telephone intervention was chosen to avoid time and travel burden for people attending face-to-face sessions and high attrition rates and low compliance to online interventions [63, 64]. Telephone-delivered CBT has been shown to be effective in people with long-term conditions, attrition rates are also significantly lower compared to face-to-face interventions [65]. Following the MRC guidelines, the current feasibility study was conducted to inform the development of a definitive full-scale effectiveness RCT. A nested qualitative component was included in the study to give contextual data and an explanation of the findings by evaluating people with IBD, therapists’ and HCPs’ perceptions of the intervention and make alterations to the CBT protocol if required to enhance its acceptability in a full-scale effectiveness trial.

The feasibility study aimed to assess the feasibility and initial estimates of potential efficacy of a CBT intervention for the management of fatigue in people with IBD. The specific research questions for the study were: (1) What is the feasibility and acceptability of a CBT intervention for the management of fatigue in people with IBD? (2) What is the feasibility of the trial protocol for delivering a full-scale pragmatic RCT? (3) What are the initial estimates of potential efficacy of an intervention for the management of fatigue in people with IBD?

## Methods

### Design

The study was a single centre, two-arm, feasibility RCT. Participants were randomised to either intervention Group 1 (CBT manual for the management of fatigue, one 60-min session and seven 30-min telephone/Skype sessions with a therapist over an 8-week period) or control Group 2 (a short fatigue information sheet to use without therapist help). A nested qualitative study evaluated patient and therapist experiences, and HCPs perceptions of the intervention. The full protocol of the study reported in this paper is published elsewhere [66]*.*

### Ethical approval

The study was granted ethical approval by the UK National Research Ethics Service - North West - Liverpool Central Committee (16/NW/0791). The trial was registered on the ISRCTN registry (17917944) on 02 September 2016.

### Patient and public involvement (PPI)

All study information, including patient information sheets, patient consent forms and questionnaire booklets, was developed with PPI to ensure acceptability and ease of understanding of what was asked of the participants. The study was approved by the hospital’s Gastroenterology Project Board Steering Committee where people with IBD, HCPs and researchers working in IBD assessed the acceptability and feasibility of the trial protocol. A group of patient and public involvement participants were mailed a draft of the intervention manual together with a feedback form in which they were asked to provide feedback on specific questions regarding the language and comprehension, format and organisation, usefulness of the manual and feasibility of the intervention. All suggested changes that made the study more acceptable to people with IBD without compromising its robustness or validity were incorporated in the manual. The Crohn’s and Colitis UK (CCUK) fatigue information sheet was developed by members of UK’s leading charity for people with CD and UC together with members of our research team (CN, WCD).

### Setting and participants

People with IBD were recruited from outpatient clinics at a single, tertiary referral, specialist hospital site between April and August 2017. Participants were included if they had a diagnosis of IBD, self-reported experiencing fatigue and were aged 18 or over. Participants were excluded if they were currently experiencing bowel symptoms they would associate with a relapse of their disease, had CBT for fatigue in the last year, were enrolled in a trial involving a non-licenced pharmacological intervention, pregnant or planning a pregnancy or were unable to give informed consent. A full list of inclusion and exclusion criteria is included in the protocol [66].

### Recruitment

A member of the patients’ direct care team at the recruitment site looked through medical records reviewing the eligibility criteria and identified potentially eligible patients attending the IBD outpatient clinic that day. At the end of their clinic appointment, the previously identified potentially eligible patients were asked by their clinician about their availability to be approached to take part in a research study. If the patients were willing to be approached, they were then provided with a participant information sheet and a full verbal explanation of the study by the study’s lead researcher. Those interested in the RCT were screened for full eligibility. If ineligible, reasons for ineligibility were recorded.

Eligible participants were given at least 48 h to consider their participation in the study. A mutually convenient time was arranged for a study researcher to answer any additional questions, verify their understanding of what the study involved and confirm their interest in study participation. Reasons for refusal to participate were recorded. Participants who agreed to take part in the study were asked to return a signed consent form and baseline questionnaires in the pre-paid stamped addressed reply envelope provided within 7 days of receipt. Screening and recruitment continued until the target sample size of 30 was reached.

### Randomisation

Randomisation was performed after participants had given informed consent and had completed and returned the baseline questionnaires. Participants were randomly allocated to one of two research arms: CBT manual plus therapist support or fatigue information sheet only. Participants were randomised at the individual level using a random number generator with a 1:1 ratio in the Statistical Package for the Social Sciences (SPSS) Version 22. The randomisation sequence was generated electronically by an independent statistician who had no patient contact prior to the commencement of the study. The trial coordinator (blinded until this point) subsequently accessed the randomisation database to assign participants to the two groups. The participants, the researchers and the therapists were not blinded to treatment allocation after randomisation. Access to usual care, including a hospital-based nurse-led helpline, was retained throughout the trial.

### CBT manual for IBD-fatigue

The CBT manual used in this trial was adapted from the CBT manual for MS-fatigue management developed by Van Kessel et al. [57] Participants received a printed copy of the manual by post. The intervention manual included a contents page, an introduction section with instructions for participants, 8 topic specific sessions and homework tasks sheets. The manual (94 pages) was presented in a transparent ring binder and each session was colour-coded to facilitate ease of use. The sessions included IBD-fatigue explained; CBT for IBD-fatigue; activity scheduling; improving your sleep; understanding IBD symptoms; changing your thinking; managing stress, determining a sense of control and coping with emotions; social support; and preparing for the future. The content of the manual was adapted with the help of one of the investigators of the original MS trials [57, 58], people with IBD-fatigue, consultant gastroenterologists and IBD-nurse specialists working with people with IBD. A medical writer and a graphic designer aided in making the language and the manual design as user-friendly as possible. Full-details of the manual development and refinement are presented in the intervention protocol [66].

### Intervention group 1 (CBT manual + therapist)

Participants in intervention Group 1 received the CBT manual for the management of fatigue; this included one 60-min and seven 30-min individual telephone sessions with a therapist over an 8-week period. The intervention support sessions were delivered by one of two qualified CBT therapists who had experience of delivering interventions to people with long-term conditions. Therapists were external, non-NHS, privately contracted and paid at the standard CBT hourly rate. The support sessions were delivered by two independent therapists to minimise allegiance bias where results are contaminated by the therapists’ experience [67]. Telephone sessions had the purpose to support the participant to collaboratively develop goals for each session using the information and resources included in the CBT manual.

### Control group 2 (fatigue information sheet only)

Participants in Group 2 received the CCUK ‘Fatigue in IBD’ Information Sheet (4 pages) to use without therapist help. Participants received the information sheet after randomisation at the same time as intervention Group 1 received the CBT manual. The information sheet provides a definition of fatigue, an explanation of what may cause it and ways to potentially reduce it (http://s3-eu-west1.amazonaws.com/files.crohnsandcolitis.org.uk/Publications/fatigue-and-IBD.pdf).

### Feasibility and acceptability outcomes

Feasibility and acceptability outcomes, their methods of assessment and progression criteria are summarised in Table 1.

**Table 1:** Feasibility and acceptability outcomes

Outcome	Objectives	Methods	Progression criteria
Recruitment	Assess the feasibility of recruiting eligible participants; assess the willingness of participants to be randomised	Recruitment log; screening log; record of reasons for participation refusal	≥ 50% of eligible participants will consent for participation in the study
Completeness of outcome data measures	Assess the completeness of the outcome data questionnaire booklets	Outcome data questionnaire booklets	≤10% missing data in each completed questionnaire booklet
Compliance	Evaluate the compliance rates of participants to the intervention	Post-intervention follow-up questionnaire with participants; semi-structured interviews with therapists	90% of participants will read all the sessions of the manual; ≥ 15 min per week will be spent completing tasks in relation to the intervention
Retention	Assess withdrawal rates during the intervention support sessions; assess the completion rates of the outcome measures at follow-up	Recruitment log therapist session log of participants’ compliance; semi-structured interviews with participants	≥ 80% of those consented will start the intervention; ≥ 70% of participants who start will complete all 8 therapist support sessions; ≤ 20% of participants will withdraw from the intervention support sessions; ≥ 70% of participants will complete baseline and 3-month follow-ups
Delivery	Evaluate therapists’ views on the intervention delivery	Semi-structured interviews with therapists	There will be positive opinions from the therapists regarding the feasibility of delivering the intervention
Implementation	Evaluate HCPs views on the intervention implementation	Semi-structured interviews with HCPs working with people with IBD	There will be support from HCPs regarding the feasibility of implementation of the intervention
Acceptability of the intervention	Evaluate participants’ experience of the intervention	Semi-structured interviews with a sub-set participants in Group 1; post-intervention follow-up questionnaire	There will be positive opinions from participants regarding an acceptably positive experience of taking part in the intervention

*IBD* inflammatory bowel disease, *HCPs* healthcare professionals

### Initial estimates of potential efficacy outcomes

Participants completed self-report questionnaires at baseline and 3, 6, and 12 months post-randomisation. Baseline measures were completed by eligible participants prior to randomisation. All questionnaire booklets were sent by post with pre-paid stamped addressed reply envelopes. At 3 months post-randomisation, participants were posted the full set of outcome measures completed at baseline, together with a post-intervention questionnaire on their experience of the intervention. To minimise participant burden, at 6 and 12 months, participants completed only disease activity, fatigue and quality of life measures.

### Baseline data

At baseline, socio-demographic and clinical data about participations were collected. Gender, age, education status, marital status, employment status and living arrangements were self-reported by participants. IBD diagnosis (CD, UC, other type of IBD), latest measurement of faecal calprotectin concentration (μg/mg), IBD-related medications (name and dose), length of time since diagnosis (months), IBD-related surgeries (number), smoking status (current smoker, ex-smoker, never smoked), exercise status (< or > 30 min of aerobic exercise per week), haemoglobin (g/dL), ferritin (nanograms per mL), serum albumin (g/L), C-reactive protein (CRP) (mg/L), platelet count (per cmm), Vitamin B12 ng/mL) and folate (nmol/mL) were retrieved from the hospital records. Clinical data are routinely collected as part of standard care. When data within 3 months before or after baseline questionnaire completion were not present, no additional blood tests were conducted, and data were marked as missing.

### Patient-centred outcome measures

Outcome measures were utilised to assess initial estimates of potential efficacy of Group 1 (CBT manual + therapist support) compared to Group 2 (fatigue information sheet only). All outcome measures were validated for self-completion. A detailed description and justification for the chosen measures is provided in the published protocol [66]. Outcome measures were sent by post with two postal reminders and/or telephone calls for non-responders after 2 and 4 weeks.

Fatigue was the primary and QoL was the secondary outcome measure for the intervention. The IBD-Fatigue (IBD-F) scale [68] was utilised to assess frequency, severity, experience and impact of fatigue. The IBD-F is an IBD-specific fatigue scale designed to identify issues of specific importance to people with IBD-fatigue. The first section of the questionnaire has five questions assessing frequency and severity of fatigue; the second section has 30 questions rating the experience and impact of fatigue. Higher scores indicate higher fatigue and higher impact of fatigue.

The UK Inflammatory Bowel Disease Questionnaire (UK IBDQ) [69] was utilised to assess IBD-specific QoL. It has 32 items, each scored in the range of 1–4, with a summary score between 30 and 120. A low score indicates poor quality of life.

The following were measured as possible explanatory variables for the intervention:

Perceptions of fatigue using the Brief Illness Perceptions Questionnaire [BIPQ] which consists of nine items: five of the items assess cognitive illness representations (consequences, timeline, personal control, treatment control and identity), two items assess emotional representation (concern and emotions) and one item assesses illness comprehensibly. Each item is rated using a response scale of 0–10; higher scores represent more threatening views of fatigue [70].

Levels of daytime sleepiness using the Epworth Sleepiness Scales [ESS]) [71]. The questionnaire asks participants to rate their chance of falling asleep or dozing on a scale of 0–3 in eight soporific situations. A total score of 0–24 is determined, with values over 10–11 indicating abnormal or pathological sleepiness.

Anxiety using the 7-item Generalised Anxiety Disorder [GAD7] scale [72], which asks participants how often during the last 2 weeks they have been bothered by each of the seven core symptoms of generalised anxiety disorder. Response options are ‘not at all’, ‘several days’, ‘more than half the days’ and ‘nearly every day’, scored as 0, 1, 2 and 3, respectively. It has a minimum possible score of 0 and a maximum possible score of 21.

Depression using the 9-item Patient Health Questionnaire [PHQ9] [73] which contains nine items, scored from 0 (not at all) to 3 (nearly every day), according to the frequency of their experience over the previous 2-week period, with a total score range of 0–27.

Disease activity: the Harvey Bradshaw Index (HBI) [74] and the Simple Clinical Colitis Activity Index (SCCAI) [75] were utilised to measure disease activity for CD and UC participants respectively.

#### Sample size

The aim of the study was not to provide a definitive estimate of treatment effect but to try out aspects of the proposed intervention for a main trial, so a formal sample size calculation was not conducted. A sample of 30 participants (15 per arm) was deemed large enough to provide useful information about feasibility based on similar feasibility studies [35, 76]. The sample was based on the same eligibility criteria that would be used in a future definitive full-scale RCT. It was recognised that the study may not have been powered to detect meaningful differences in clinically important endpoints [77].

#### Statistical analysis

The feasibility of recruiting participants and willingness to be randomised was evaluated by calculating the proportions of those invited for participation in the study who were eligible and who decided to take part in the trial, together with reasons for ineligibility and participation refusal. The completeness of data collected was assessed by reviewing the proportion of pages and proportion of items of the outcome measure booklets that were completed by the participants. Concordance rates were assessed through a post-intervention follow-up participant questionnaire asking participants about the number of sessions of the manual they read, the number of telephone therapist support sessions they completed and the time per week they spent completing tasks in relation to the intervention. Therapists were instructed to keep a log recording how many sessions were completed by each participant, the frequency of the sessions and participant interruptions or withdrawals from the interventions. Completion rates of baseline and outcome measures at 3, 6 and 12 months post-randomisation were recorded.

Descriptive data were calculated presenting means and standard deviations for all continuous data and frequencies and percentages for categorical variables at baseline, 3, 6 and 12 months post-randomisation. Analysis to determine initial estimates of potential efficacy for the primary and secondary outcomes was conducted on ‘complete cases’, comparing intervention Group 1 and control Group 2 only for participants who completed both baseline and one or more of the follow-ups [78]. The difference in mean change between baseline and 3 months, 6 months, and 12 months’ follow-up was conducted for the primary (IBD-F) and secondary (IBDQ) outcomes. Following guidelines suggesting that feasibility studies focus on estimation rather than significance testing [79–81], effect sizes and 95% confidence intervals were also presented. A power analysis was performed in order to calculate how many participants would have to be included in a potential future trial in order to have 90% power to find a difference at the level of *p* = 0.05 for the fatigue severity subscale of the IBD-F.

#### Nested qualitative study

Feasibility of delivering the intervention was assessed by conducting semi-structured interviews with the therapists. Both therapists supporting participants during the intervention were interviewed at the end of their intervention delivery. Feasibility of implementation of the intervention within the existing IBD service at the study site was assessed by conducting semi-structured interviews with HCPs working with people with IBD. HCPs were interviewed after recruitment completion. All HCPs working with people with IBD at the study site were offered an interview by the lead study researcher, purposive sampling was utilised to recruit a minimum of one HCP in each role (consultant gastroenterologist, specialist registrar in gastroenterology, IBD nurse specialist) until data saturation was reached.

Acceptability of the intervention to participants was assessed by conducting semi-structured interviews with a sub-set of participants in intervention Group 1. Semi-structured interviews using an interview topic guide were conducted with a sub-set of approximately one third of participants in Group 1 (CBT + therapist support). The participants were purposively selected to include if possible both genders, a range of ages, both IBD diagnoses and undertaking telephone sessions with both therapists. Furthermore, in the post-intervention follow-up questionnaire all participants in Group 1 were asked about their preferences for the format and delivery, their satisfaction and comments on the intervention.

The nested qualitative study was conducted after the 3-month follow-up quantitative data collection point. Interviews were conducted face to face or over the telephone by a researcher not involved in the delivery of the intervention. Interviews were digitally audio-recorded, anonymised, and transcribed verbatim by a professional transcriber. Two researchers (MA, HP) analysed the data, one of whom (MA) conducted the interviews. Data were analysed using deductive thematic analysis [82]. A pre-existing coding framework was developed utilising the study objectives and the interview schedules in order to answer the specific research questions of the study. Researchers analysed all transcripts independently utilising the same coding framework. Resulting themes were then compared and any differences were resolved by discussion. Themes were then refined prior to producing the final report of key themes and sub themes.

## Results

### Baseline characteristics

Baseline characteristics of the 31 consented participants are summarised in Table 2.

**Table 2:** RCT patient participants’ baseline characteristics

Variable, *N* (%) unless otherwise specified	Intervention Group 1 (15)	Control Group 2 (16)
Female gender	10 (67)	10 (62)
Age, mean (range), years	37.00 (31)	39.13 (33)
Education		
Up to 16	1 (6.6)	2 (12.5)
Up to 18	0	1 (6.2)
Higher education	14 (93.3)	13 (81.2)
Marital status		
Married/living with partner	9 (60)	11 (68.7)
Widowed/divorced	2 (13.3)	0
Single/single parent/Other	4 (26.7)	5 (31.2)
Employment status		
Full time/part time	14 (93.3)	13 (81.2)
Retired	0	0
Not working/housekeeping	1 (6.6)	3 (18.8)
Living status		
Alone	2 (13.3)	3 (18.8)
With partner/spouse/child/ other relatives/friends	13 (86.7)	13 (81.2)
Smoking status		
Yes	0	1 (6.2)
Ex-smoker	5 (33.3)	9 (56.2)
No	10 (66.7)	6 (37.5)
Exercise status (weekly)		
> 30 min aerobic exercise	12 (80)	10 (62.5)
< 30 min aerobic exercise	3 (20)	6 (37.5)
Disease classification		
UC	3 (20)	4 (25)
CD	11 (73.4)	10 (62.5)
IBDU	1 (6.6)	2 (12.5)
CD Montreal classification (*n =* 20)		
L1 ileal	6 (60)	3 (30)
L2 colonic	0	4 (40)
L3 ileocolonic	4 (40)	3 (30)
Months since diagnosis, median (range)	171.13 (879)	217.00 (972)
Current medication (yes) *n* (%)	10 (67)	14 (87)
Thiopurines	6	6
Methotrexate	0	1
Anti-TNF	7	4
Vedolizumab	0	2
Steroids	0	1
Previous IBD surgery (yes) *n* (%)	1 (6.67)	4 (25)
Current stoma (yes) *n* (%)	0	1 (6.2)

*anti-TNF* Anti-Tumour Necrosis Factor, *CD* Crohn’s Disease, *IBD* Inflammatory Bowel Disease, *IBDU* inflammatory bowel disease unclassified, *UC* ulcerative colitis

#### Feasibility and acceptability outcomes

##### Recruitment

**Fig. 1:**
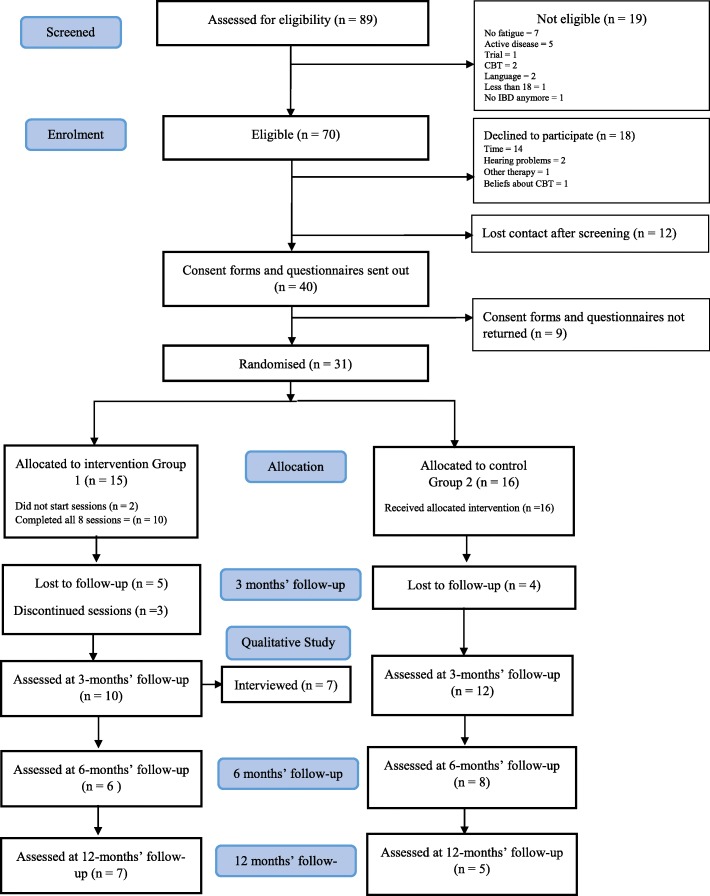
Consort diagram of patient flow

A total of 89 consecutive people with IBD were referred to the intervention and approached for participation. Seventy of these were eligible and 31 consented to participate, giving a recruitment rate of 44%. A Consort diagram of patient flow is presented in Fig. 1. Of the 39 who did not consent for the study, 18 declined to participate and for another 12 contact was lost after initial screening. The main reason for declining to participate was the time commitment required for the study (78%). Other reasons included hearing problems (11%), currently undergoing other psychological therapy (5.5%) and negative beliefs about CBT (5.5%).

### Completeness of outcome data measures

The average percentage of missing data in each completed baseline and 3 months’ follow-up questionnaire booklet was 3.7%. The scale with the highest percentage of missing data was the IBDF-3. There were no missing data in the 6 and 12 months’ follow-up questionnaire booklets received.

### Compliance

Participants’ post-intervention questionnaires (*n* = 10) indicated 9 (90%) participants read all the sessions of the manual and 8 (80%) completed the homework tasks. Three (30%) participants spent more than 90 min, 2 (20%) spent 60–90 min and 5 (50%) spent 30–59 min per week completing the intervention.

### Retention

Of the 15 participants who consented and were randomised to the intervention group, 13 started the intervention (87%). The therapists’ log of participants’ sessions indicated that 10 (76.9%) of those who started completed all telephone sessions over 8 weeks. Two (13.3%) discontinued the intervention prior to commencing the telephone sessions. One participant discontinued due to work schedule interference and one participant due to a newly diagnosed unrelated illness. Three (20%) discontinued the intervention after commencing the telephone sessions. One participant discontinued due to time commitments at Session 1, one due to family illness at Session 2, and one due to perceived un-usefulness of the intervention for their needs at Session 4. Ten (66.7%) participants in intervention Group 1, 12 (75%) in control Group 2 and 22 (71%) participants overall completed baseline and 3 months’ follow-up questionnaires. Six (40%) participants in intervention Group 1, 8 (50%) in control Group 2 and 14 (45.2%) overall completed 6 months’ follow-up questionnaires. Seven (46.7%) participants in intervention Group 1, 5 (31.3%) in control Group 2 and 12 (38.7%) overall completed 12 months’ follow-up questionnaires.

### Acceptability of the interventions: participants’ interviews

#### Post-intervention follow-up survey

The mean satisfaction score from 0 to 10 (*n* = 10) for intervention Group 1 was 8.6 at 3 months’ follow-up. All participants reported they would continue using the strategies learned in the intervention. For future iterations of the intervention, six (60%) participants reported they would prefer to complete the intervention online and 4 (40%) participants reported they would prefer to complete the sessions face-to-face instead of over the telephone.

The mean satisfaction score from 0 to 10 (*n* = 9) for control Group 2 was 2.4. Participants spent a mean of 9.6 min reading the fatigue information sheet. Most of the participants did not find the fatigue information sheet useful as they perceived it did not provide them with any additional strategies to manage fatigue they were not already aware of.

#### Semi-structured interviews with participants

Of the 15 participants who were randomised to Group 1 of the feasibility RCT, 7 were interviewed. Four participants were women and their median age was 40 (range = 19). The majority of participants had CD (*n* = 5), one had UC, and one IBD-U. Six participants had completed all 8 sessions and one participant had completed 4 sessions. The main themes that emerged from the interviews were ‘Outcomes of the intervention’, ‘Views on the intervention manual and the telephone sessions’, ‘Format of the intervention’ and ‘Suggestions for improvement’. All of the major themes had interlinked sub themes. The foundations of these themes are described alongside verbatim quotes to illustrate them. Participants are identified in brackets after each quote by using codes for IBD disease type, gender and age.

1. Outcomes of the intervention

- Impact of the intervention

Feedback on the intervention was mostly positive. Most participants believed that taking part was a worthwhile and valuable experience and that they would recommend it to other people. They reported that the skills they learned positively impacted on their fatigue and their lives to varying degrees. Some participants specifically mentioned feeling disappointed upon completion of the telephone sessions and wishing they would continue for longer.

I got quite upset and quite emotional, not upset upset, but emotional about it, because like this has really benefited me and I’ve really, I really enjoyed doing it and it really benefited me. And I cannot underestimate how, yes, how much it was so useful. I do genuinely feel so much better now (IBDU_F29)

(b) Useful knowledge/skills acquired with the intervention

As a result of the intervention, participants learned more about fatigue and its causes. They also mentioned specific examples of useful knowledge/skills they acquired which led to improvements in their fatigue and/or quality of life. Almost all participants talked about the negative all-or-nothing behavioural patterns they had been adopting before the trial, and how the intervention had taught them to better pace their activity levels through self-monitoring, introducing breaks, planning their time effectively and learning to say no.

I would just try and ignore it as best I can and basically get on with it and try and rush and get everything done before it came to the point I had to stop. Where now I do try and, if you like, pace myself a little bit more. So, it’s, I can actually get a little bit more done (CD_M48)

Participants also often referred to a change in the way they thought and felt about their fatigue, highlighting that having more positive thoughts about fatigue had helped them feel better about it, manage it more effectively and ensure it had a lesser impact on their lives.

A good part of the study, I found, was trying to focus on different ways to think and I was doing that over a kind of period of it. And it certainly helped (CD_M44)

Two participants emphasised how the intervention had aided them to improve their sleep quality, ultimately leading them to have more energy during the day.

I am getting a better night’s sleep. I am having a little bit more energy during the day. I am getting a bit more done … (CD_F44)

Finally, the same two explained how the intervention had prompted an increase in their physical activity levels.

I now walk into work now … But I also, in the weekend, I will go for a 40-45-minute walk as well just to – I want to keep on building on it (IBDU_F29)

2. Views on the intervention manual and the telephone sessions

- Comprehensibility, structure and completeness of the manual

The information the in the manual was perceived to be clear and easy to understand. The content of the manual and the telephone sessions was considered to be well-planned. The manual was thought to be comprehensive and all the topics covered were considered relevant and necessary. Although two participants sometimes found it hard to fit the completion of the homework tasks and one did not find certain parts of the homework relevant to him, all of the participants acknowledged their importance as enablers to the interventions’ positive outcomes.

I found doing the homework sometimes a little bit of a challenge … but I wouldn’t say I wish it wasn’t there, because, unless I did it, I wouldn’t have got the most out of the process (CD_F31)

(b) Length, number and intervals between the therapist support sessions

Aside from one participant who thought they were the right length, the majority found the 30-min telephone sessions to be rushed and would have preferred for them to be longer. One participant suggested merging the two sessions on thoughts and emotions, another recommended the addition of a ninth session that could summarise the content of the intervention and give the opportunity to ask questions and refresh people’s memories on the skills they had previously acquired. Not all participants agreed on the intervals between sessions. According to one participant, having sessions once a week worked well because it helped to develop a routine and provide a structure for the completion of the homework tasks. The other two participants believed 1 week in between sessions was not long enough and having two weekly or monthly sessions would have allowed for more time to reflect on the material and the better identification of behavioural trends through activity monitoring.

A week is just not long enough. A month would probably be the ideal period, to be honest (CD_M44)

3. Format of the intervention

- Relative importance of the manual versus the telephone sessions

Everyone agreed on the importance of having the manual together with the therapy sessions, as the two complemented each other. The manual provided the participants with useful information they could refer back to in between and after the sessions were completed. Equally, the therapist added to the intervention by explaining the concepts and tailoring examples to participants’ needs.

Until somebody explains what that tool does, it’s just words on a page. And that was part of what the therapist was doing, she was explaining why that particular tool, why that particular method would work … So it’s not just a process, words on a page, it’s something tangible, something that is going to give you a net benefit (CD_M44)

(b) Modality of communication of the therapy sessions

Responses to the use of the telephone were all positive. The main reported advantage of conducting sessions over the phone was the convenience of not having to travel to the hospital and being able to fit in the sessions during their work day.

I actually really, I enjoyed it and I found it very convenient (IBDU_F29)

(c) Possibility of an online intervention

Views on the possibility of an online intervention were mixed. Participants believed that having the manual in electronic format would be feasible and potentially beneficial for the completion of the homework tasks. However, they recognised the value of having a therapist to support the online intervention in order to guide the sessions, answer questions and ensure compliance with the intervention.

Part of what made me really invest in the process was talking to the therapist every week, and having her understand. And having her listen and encourage and support. And you just can’t get that from a computer (CD_M31)

4. Suggestions for improvement

Referring to the intervention manual, participants suggested moving the homework tasks to the end of each corresponding session instead of at the end of the manual, having the option to complete the homework tasks through dictation, adding examples of things other than fatigue where the intervention strategies could be applied, and changing the name of the intervention manual to MODIFY Fatigue in order to further emphasise its psychological aspects. With regard to the telephone sessions, participants suggested having someone who had experienced fatigue deliver the intervention and involving a trusted confidant in the last session about social support. Finally, two of the participants, one who dropped out at session 4 and one who completed all eight sessions, advised for a higher threshold of fatigue to be set when enrolling participants in the intervention. Not having experienced negative thoughts associated with fatigue and having already put in place the necessary coping strategies to deal with it, they felt that the intervention was not suitable for them. They suggested the intervention may be more useful for people with more severe fatigue or those who have been newly diagnosed and therefore would need to learn strategies to manage the impact of the disease on their lives.

It would be something that I would have really liked to have had when I was first diagnosed... I’m not sure like now is the right time for me to, for me to have been doing it, because it’s, yes, I’m just not in the kind of place, I think, most of the people who are doing this, were (UC_F31)

### Feasibility of delivery: therapists’ interviews

Both of the therapists delivering the intervention to Group 1 were interviewed to enquire about their opinions on the feasibility of delivering the intervention. The themes mimicked the ones emerging from participants’ interviews.

#### Outcomes of the intervention

The therapists had a very positive experience of delivering the intervention. They believed that there was a need for this type of intervention for people with IBD-fatigue and that this intervention equipped people with skills they could apply to self-help in the future. For the therapists, the most useful components of the intervention for the patients were changing the way they thought about fatigue, recognising the importance of the psychological aspects of fatigue, realising that they were not alone in their suffering from fatigue and learning to monitor and schedule their activities more effectively.

Towards the end, whether knowingly or not, I don’t know, they would start, you could see that kind of their perspectives had changed slightly. And they’d start rephrasing things or talking about things in a slightly different way without being prompted (Therapist 1)

Although according to one therapist the intervention could also be adapted for a more moderate severity, both therapists thought that the intervention was more appropriate for people who experienced more severe levels of fatigue. Furthermore, one therapist stressed the importance of offering the therapy only to people seeking help for fatigue and the other suggested it could benefit people with IBD at the point of diagnosis.

If people are afflicted with the fatigue and it has an impact, they’re potentially more motivated than someone who maybe isn’t experiencing the fatigue as badly (Therapist 1)

#### Views on the intervention manual and the telephone sessions

The manual was perceived to be a good resource for the therapists and the participants. Whilst one therapist found the colour coding of the pages of the sessions useful, the other found it off-putting and would have preferred the use of coloured index tabs. Both therapists also suggested to move the homework tasks the end each session, instead of having a homework booklet at the end of the manual. Additionally, one therapist advised it would be beneficial for patients to continue their activity monitoring for 2 weeks instead of only for 1 week.

The therapists agreed that the 30-min sessions were too short and would have preferred the sessions to be at least 45 min. Whilst one therapist considered the number of sessions to be appropriate, the other would have preferred to add an additional ninth session on thoughts. Moreover, it was suggested either switching the order of the sessions to bring the one on thoughts forward or having the option to keep the order of the sessions flexible so it could be tailored to participants’ needs.

#### Format of the intervention

Although the therapists were initially concerned about not being able to pick-up non-verbal cues over the phone, they both agreed that anonymity of the phone may have allowed participants to self-disclose more than if the sessions had been face-to-face. However, one therapist suggested that face-to-face sessions would have pushed some participants to get out of bed and the other believed that having the first session face-to-face could have made participants more vested in the process.

I think it was – it normalised it nearly, because you have phone conversations all the time (Therapist 1).

There was disagreement on the relative importance of the manual and the therapists’ sessions. One therapist thought that the manual could be utilised by participants on their own and that they might only struggle on more complex topics such as ‘thought-challenging’. The other was adamant that a therapist was necessary to guide them through the sessions.

They need to have access to somebody who’s going to coach them through it (Therapist 2).

Both therapists agreed that although no prior experience with people with IBD was needed to deliver the intervention, it would have been useful to have a better knowledge of the physical symptoms experienced by people with IBD and the medications utilised for its management. According to one therapist, a CBT therapist with experience in fatigue would be the ideal HCP to deliver the intervention, yet an IBD-nurse specialist could potentially deliver it with adequate CBT training.

I think they would nearly be more, I don’t want to say ‘qualified’, that’s the wrong term, but in a better place to deliver it with some training about kind of CBT techniques and Socratic questioning and all those kind of things, than potentially a CBT therapist or a clinical psychologist who had very, very little experience of IBD (Therapist 1)

Taking into account barriers such as computer illiteracy and difficulty in accessing the online information once the intervention has ended, one therapist believed that on online intervention could be beneficial in providing a more tailored experience for the participants.

#### Suggestions for improvement

The therapists advised other suggestions for improvement of the intervention, including: adding more real-life examples in the manual, utilising the term ‘home practice’ instead of ‘homework’, having an online booking system for appointments, sending photos of the homework tasks to the therapist through a secure transfer method and organising the opportunity for people with IBD-fatigue to share their experiences between them (online and/or in person).

### Feasibility of implementation: HCPs’ interviews

Four HCP (one consultant gastroenterologist [CG], one IBD-nurse specialist [CNS] and two IBD research fellows [RF]) were interviewed to enquire about their opinions on the feasibility of implementing the intervention in their IBD service. The main themes that emerged from the interviews were ‘Benefits of the intervention’ and ‘Barriers to the intervention’.

#### Benefits of the intervention

Although doctors reported routinely enquiring about other IBD-related symptoms, they reported only discussing fatigue if the patient brought it up. Indeed, both doctors and the nurse acknowledged that having a conversation about fatigue may add time to the consultation, making it difficult to find the time to talk about it in detail. Indeed, all HCPs reported struggling to help people with IBD with the understanding and management of fatigue and wanting to offer them a solution for their fatigue. Furthermore, the need for a psychological support service for people with IBD was expressed. HCPs felt that if a psychologist was employed to deliver the fatigue management intervention they could also support people for other psychological problems which are not currently addressed by the service.

As clinicians, it would be a good way of solving a problem that currently we can’t solve, or, at least offering a possible solution, because we don’t have much to offer them (CG).

HCPs found the intervention to be comprehensive and useful, and they appreciated its structure in separate sections. They thought the applicability of the intervention was quite broad. One doctor reported initially being sceptical about participants’ response to the intervention, yet he subsequently found participants to be keen to be involved.

I think my first thought was that patients are going to go, “Well I’m not making it up, if you think I’m making it up.” But they haven’t actually. They’re very keen on it and they’re very keen to be involved. And they thought it was a very good idea (RF02)

Likewise, another doctor reported recruitment for the intervention to be straightforward. Two HCPs agreed they would offer the intervention to anyone in remission. Additionally, the nurse suggested that certain aspects of the intervention may benefit also people with active disease.

#### Barriers to the intervention

Time, training and financial resources were found to be barriers to the implementation of the intervention. All HCPs reported time being a barrier. The four and a half hours of one-on-one time needed for the telephone sessions were recognised as a significant amount of time if the number of people interested in the intervention was expected to grow. Both doctors and the nurse acknowledged that the nurses would not have the time to deliver the intervention and that if they made the time, other aspects of the service would suffer. Likewise, no one else in the current team was seen as having the time to offer the intervention to all people with IBD experiencing fatigue.

There is no way that our nursing service could take on that burden (CG)

HCPs all agreed that an additional HCP would therefore have to be employed specifically to oversee the intervention. Alternatively, either the intervention manual would have to be given to participants without the support sessions or an internet platform/app would have to be designed for the participants to self-manage their fatigue.

If there’s an internet platform or an app of some description that they could do the necessary exercises and take them through the process without needing someone else to guide them, that would be, you know, that would be ideal (RF02)

The nurse believed that the nurses would benefit from having adequate training on IBD-fatigue management. However, she did not have a clear idea of what the training would look like. Lastly, all the doctors acknowledged the need for taking the cost of the one-on-one support sessions and funding into account when considering the implementation of the intervention. The doctors suggested different ways in which cost-effectiveness of the intervention could be tested. One interviewee suggested that addressing fatigue and other psychological problems may in turn improve IBD and save money in the long-run. More specifically, the consultant argued that cost-effectiveness could be assessed and shown through reductions in outpatient appointments in a trial.

There may be an argument, you know, with certain patients, tacking their fatigue or whatever other psychological problems they may have. You may be able to intervene and get them medically better, which may save money in the long term (RF01)

#### Initial estimates of potential efficacy outcomes

Table 3 shows means and standard deviations for outcome measures at baseline, 3, 6 and 12 months post-randomisation by treatment group.

**Table 3:** Means and standard deviations of outcome measures of all participants at baseline, 3, 6 and 12 months’ follow-up measures

Outcome	Group	Baseline			3-month follow-up			6-month follow-up			12-month follow-up		
		Mean	SD	*N*	Mean	SD	*N*	Mean	SD	*N*	Mean	SD	*N*
HBI	Group 1	3.30	3.2	10	5.50	6.05	8	11.20	5.45	5	8.00	2.45	5
	Group 2	4.33	2.74	9	5.50	4.46	6	7.80	3.70	5	5.00	0.00	3
SCCAI	Group 1	5.22	3.22	3	4.00	2.83	2	7	0	1	11.00	4.24	2
	Group 2	4.50	2.52	4	4.50	1.22	6	8.67	1.16	3	7.50	0.71	2
IBD-F1	Group 1	11.93	3.24	14	8.00	4.61	9	7.83	3.66	6	8.43	2.51	7
	Group 2	9.93	4.13	15	9.45	4.97	11	8.13	4.16	8	8.00	2.65	5
IBD-F2	Group 1	55.83	26.74	12	23.40	15.09	10	31.75	25.86	6	29.85	18.89	7
	Group 2	49.00	28.66	15	44.20	30.96	10	23.49	25.33	8	21.05	12.66	5
IBDQ	Group 1	89.67	13.67	15	97.50	11.20	10	91.83	18.70	6	97.57	8.89	7
	Group 2	93.79	8.87	14	95.27	10.10	11	100.88	10.86	8	100.00	4.30	5
BIPQ	Group 1	41.13	6.20	15	32.40	6.83	10						
	Group 2	42.40	7.14	15	43.20	6.14	10						
ESS	Group 1	13.87	4.85	15	7.00	4.42	10						
	Group 2	10.19	4.69	16	9.82	3.90	11						
GAD7	Group 1	9.80	5.54	15	4.50	3.53	10						
	Group 2	8.00	5.27	16	5.00	3.52	11						
PHQ9	Group 1	12.00	6.01	15	6.00	4.42	10						
	Group 2	9.75	6.00	16	8.73	4.71	11						

*BIPQ* Brief Illness Perceptions Questionnaire, *ESS* Epworth Sleepiness Scale, *GAD* generalised anxiety disorder, *HBI* Harvey Bradshaw Index, *IBD-F* Inflammatory Bowel Disease-Fatigue, *IBDQ* Inflammatory Bowel Disease Questionnaire, *PHQ* Patient Health Questionnaire, *SCCAI* Simple Clinical Colitis Activity Index, *SD* standard deviation

#### Three months’ follow-up

Table 4 shows means, standard deviations, change scores and effect sizes of participants who completed baseline and 3 months’ follow-up primary and secondary outcome measures.

**Table 4:** Means, standard deviations, change scores and effect sizes of participants who completed baseline and 3 months’ follow-up primary and secondary outcome measures

Outcome	Group	Baseline		3 months’ follow-up			Change scores			Between group effect sizes (CI)
		Mean	SD	Mean	SD	*N*	Mean	SD	MD (CI)	
IBDF-1	Group 1	11.12	3.56	7.00	3.74	8	− 4.12	4.91	− 2.94 (− 7.21, 1.32)	0.84 (− 0.5, 1.82)
	Group 2	10.63	4.25	9.45	4.87	11	− 1.18	2.28		
IBDF-2	Group 1	53.25	31.67	23.00	16.38	8	− 30.25	23.90	− 26.14 (− 29.30, − 2.98)	1.20 (0.13, 2.27)
	Group 2	51.44	33.24	47.33	31.11	9	− 4.11	20.08		
IBDQ	Group 1	90.80	15.08	97.50	11.20	10	6.70	11.09	2.70 (− 6.45, 11.85)	− 0.25 (− 1.21, 0.72)
	Group 2	91.70	9.79	95.70	10.54	10	4.40	8.01		

*CI* confidence interval, *IBD-F* Inflammatory Bowel Disease-Fatigue, *IBDQ* Inflammatory Bowel Disease Questionnaire *MD* mean difference, *SD* standard deviation

There was a reduction in IBD-F fatigue severity and fatigue impact scores in both groups. Participants in intervention Group 1 showed a mean change score of − 4.12 (SD = − 4.91) at 3 months compared to baseline for fatigue severity (IBD-F1). Participants in control Group 2 showed a mean change score of − 1.18 (SD = − 2.28) at 3 months compared to baseline. The mean difference (MD) between the change scores in Group 1 and Group 2 was − 2.94 (confidence intervals [CI] = − 7.21, 1.32), with a between group effect size of 0.84 (CI = − 0.5, 1.82).

Participants in intervention Group 1 showed a mean change score of − 30.25 (SD = 23.90) at 3 months compared to baseline for fatigue impact (IBD-F2). Participants in control Group 2 showed a mean change score of − 4.11 (SD = − 20.08) at 3 months compared to baseline. The MD between change scores in Group 1 and Group 2 was − 26.14 (CI = − 29.30, − 2.98), with a between group effect size of 1.20 (CI = 0.13, 2.27).

There was an improvement in quality of life (IBDQ) in both groups. Participants in Group 1 showed a mean change score of 6.70 (SD = 11.09) at 3 months compared to baseline. Participants in Group 2 showed a mean change score of 4.40 (SD = 8.01) at 3 months compared to baseline. The MD between change scores in Group 1 and Group 2 was 2.70 (CI = − 6.45, 11.85), with a between group effect size of − 0.25 (CI = − 1.21, 0.72).

#### Six and 12 months’ follow-up

Additional file 1: Table S1 shows means, standard deviations, change scores and effect sizes of participants who completed baseline and 6 months’ follow-up primary and secondary outcome measures.

The reduction in fatigue severity and fatigue impact scores was maintained in both groups. There was still a greater change between baseline and 6 months in fatigue severity (IBD-F1) in intervention Group 1 than control Group 2 (MD = − 2.90 [CI = −7.39, − 6.88)]), with a between group effect size of 0.83 (CI = − 0.27, 1.93). Likewise, the MD between change scores in fatigue impact (IBD-F2) in Group 1 and Group 2 was − 15.59 (CI = − 31.55, − 39.99), with a between group effect size of 1.30 (CI = 0.14, 2.47).

There was an improvement in quality of life (IBDQ) in both groups. However, control Group 2 showed greater change between baseline and 6 months compared to intervention Group 1 (MD = − 2.91 [CI = − 15.18, 10.50]), with a between group effect size of 0.23 (CI = − 0.83, 1.29).

Additional file 1: Table S2 shows means, standard deviations, change scores and effect sizes of participants who completed baseline and 12 months’ follow-up primary and secondary outcome measures.

The reduction in fatigue severity scores was maintained in both groups. There was a still greater change between baseline and 12 months in fatigue severity (IBD-F1) in intervention Group 1 than control Group 2 (MD = − 1.77 [CI = − 4.62, 1.10)]), with a between group effect size of 0.91 (CI = − 0.30, 2.11). The reduction in fatigue impact scores was partly maintained in intervention Group 1 but not in control Group 2, with a MD in change scores of − 14.53 (CI = − 39.55, 10.49) and a between group effect size of 0.87 (CI = (− 0.22, 2.07).

There was an improvement in quality of life (IBDQ) in both groups. In contrast with the 6 months’ follow-up, intervention Group 1 showed greater change between baseline and 12 months compared to control Group 2 (MD = 8.43 [CI = − 1.74, 18.60]), with a between group effect size of 0.98 (CI = − 2.10, 0.23).

## Discussion

This feasibility study aimed to evaluate the feasibility and initial estimates of potential efficacy of a CBT intervention for fatigue in people with IBD. Quantitative and qualitative methods were utilised to determine whether the progression criteria for continuation to a full-scale effectiveness trial were met. Feasibility progression criteria assessing completeness of outcome measures and compliance were met; those of recruitment and retention were not fully achieved. The intervention was acceptable to participants and feasible for the CBT therapists to deliver. However, HCPs identified potential barriers to the feasibility of implementation of the intervention.

Preliminary efficacy analysis based on a small number of complete cases showed there was a greater reduction at 3, 6 and 12 months post-randomisation in severity and impact of fatigue for intervention Group 1 compared to control Group 2, with large between group effect sizes. There were greater improvements in QoL for intervention Group 1 compared to control Group 2 at 3 and 12 months’ follow-up, but not at 6 months’ follow-up. Overall these findings suggest a full-scale effectiveness RCT testing CBT for IBD-fatigue is feasible and potentially worthwhile with some changes to the protocol.

### Advised revisions to the protocol

The baseline and 3 months’ follow-up questionnaires returned by participants had an average of 3.7% missing item responses suggesting that the measures used were acceptable to participants [83]. The use of mobile apps for questionnaire completion in the full-scale trial could nonetheless further improve data completeness compared to using paper questionnaires [84]. Ninety percent of participants were compliant by reading all the sessions in the manual and all of them reported spending more than 15 min per week performing the intervention tasks. Furthermore, although 13% of participants randomised to intervention Group 1 did not start the therapist support sessions, 77% of those who started completed all 8. Compared to the original intervention for MS-fatigue [57] where all participants completed 100% of the sessions, this may suggest first treatment exposure to be a central treatment component of CBT [85]. Indeed, having completed at least one session versus to not having completed any sessions is a strong predictor of engagement and long-term symptom reduction in CBT trials. Including strategies to improve engagement at the beginning of the intervention may therefore be a key feature to incorporate in the full-scale trial [86]. Telephone sessions, which were very positively received by the participants, may also have contributed to the high compliance rates. Telephone delivery can reduce the geographical barriers associated with face-to-face therapy [87] and more interestingly it may overcome patient ambivalence towards psychological treatment [88], ultimately reducing attrition [89]. The inclusion of telephone sessions should thus be included in the full-scale trial.

Although recruitment target for participants was achieved, 44% of eligible participants consented for participation instead of 50% as defined a priori. The main reason for declining to participate was the time commitment required for the intervention, indicating time required from participants may be a potential barrier to the uptake of a full-scale CBT trial [90]. Online intervention modalities which increase flexibility for participants in regard to time and location of accessing treatment [91] should thus be considered to incentivise participation. Conversely, as lack of motivation to take part has been posited as a reason for drop-out from trials [92], it is nevertheless important to recruit those participants who are motivated to participate in the CBT trial because their fatigue is burdensome. Findings from our interviews showed that HCPs rarely ask about fatigue and not all participants mention it during outpatient consultations, making recruitment of participants experiencing fatigue through clinician referral difficult.

The withdrawal rate during treatment of 23% and reasons for withdrawal are comparable to meta-analytic findings on CBT drop-out rates across conditions [93]. Nonetheless, withdrawal rates were slightly higher than the 20% initially anticipated for progression to the full-scale RCT. Likewise, at 3 months’ follow-up, 4 out of the 16 participants in control Group 2 and 5 out of the 15 participants in intervention Group 1 did not return the completed questionnaires despite the two reminders. The loss to follow-up rate further increased at 6 (Group 1 = 60%, Group 2 = 54.8%) and 12 months’ follow-up (Group 1 = 68.7%, Group 2 = 61.3%). One possible reason for these higher than expected loss to follow-up rates may be associated with participants’ perception of subjective benefits as a result of the intervention. When participants perceive that they have experienced positive changes they may be more motivated to comply with the trial procedures [94]. Conversely participants who have withdrawn from the support sessions or those have been randomised to the control group may be less invested in returning the follow-up questionnaires [95]. Stratification by fatigue level might address any potential for those with worse fatigue to preferentially drop-out and even out any loss to follow-up across randomised groups.

Unfortunately, blinding of participants is impossible in psychosocial interventions [96]. However, further testing a trial design for the full-scale RCT which includes a comparison instead of a control group and in which intervention time is kept constant across the two groups may help to minimise the effect of participants’ expectancy on attrition [97]. Additionally, blinding of researchers (rather than participants) should be possible.

Most participants in intervention Group 1 provided positive opinions regarding their experience of taking part in the intervention. Participants believed that the intervention manual complemented the therapist support sessions and they reported changes in patterns of negative feelings, cognitions and emotions in line with the CBT aims. The therapists reported that they would use this intervention for the management of IBD-fatigue so as to equip participants with skills to self-help. However, both the participants and CBT therapists agreed that the intervention was more useful for people with higher fatigue levels. Assessment of higher levels of fatigue utilising standardised fatigue measures should therefore be included when screening for eligibility in the full-scale RCT. Additional changes suggested by the participants and CBT therapists to improve feasibility and acceptability of the intervention included increasing the length of the telephone sessions, having the homework tasks at the end of each corresponding session, using an online booking system to book appointments and sending photos of homework tasks to the therapists to ensure compliance. These suggestions should feed into future intervention development.

Despite HCPs finding a broad applicability and perceived utility of the intervention to their IBD patient group, they identified potential time, training and financial barriers to the feasibility of implementation of the intervention within their current IBD service. No current member of their IBD team was seen to have enough time and adequate training in CBT to deliver the intervention; specific training with protected time would therefore have to be provided. The presence of an economic evaluation of the intervention to demonstrate its cost-effectiveness is consequently important in the full-scale RCT. Additionally, online interventions with therapist support sessions may have the potential to reduce demand on clinicians and lower costs whilst still maintaining a personalised approach to participants and controlling attrition [98]. Indeed, as CBT manuals are characterised by being systematic and operationalised [99], they can translate well into computerised interventions [100]. Likewise, whilst tailoring therapy to individual needs is more resource intensive to develop, it might yield better outcomes over time than entirely self-directed online therapies [64].

### Sample size calculation

To detect a mean difference in IBD-F severity scores at 12 months post-randomisation with a two-sided significance level of 5% and a power of 90% with equal allocation to two arms would require 61 participants in each arm of the trial. To allow for a drop-out of approximately 29% (finding from this study) at 3 months post-randomisation and a 10% drop-out at both 6 and 12 months post-randomisation, then a minimum of 107 people with IBD should be recruited per arm (214 in total). Given that in this feasibility study 31 participants were recruited from a single centre over 5 months, these 214 participants could potentially be recruited from 5 to 10 centres over a year of recruitment. Based on our experience it is likely that three times the number required would need to be screened for eligibility.

### Strengths and limitations

Development of the intervention was guided by the MRC framework for complex interventions [61], based on cognitive-behavioural theory and empirical evidence in IBD and other long-term conditions. Extensive consultation with people with IBD was conducted in order to make the intervention more relevant and acceptable to the target population. A series of steps were taken to minimise bias or systematic errors and to improve trial rigour: the randomisation sequence was generated by an independent statistician who was not involved in the operating of the trial to avoid contamination; interviews were conducted after the quantitative data collection to avoid influencing participants’ experience of the treatment [101] and analysed by two independent researchers to avoid researchers’ biases and enhance confidence in the findings [102, 103].

Nonetheless, analysis of pilot and feasibility studies should be mainly descriptive resulting in preliminary findings which should be tested in large-scale effectiveness RCTs [79]. The sample was small and limited to people attending one tertiary referral centre and cannot therefore be generalised to the wider population of people with IBD. The use of patient self-report disease activity scores may have resulted in incomplete or inaccurate data on remission status. Although self-reported disease activity scores are often utilised in research studies, it is best practice for clinicians to complete them after obtaining information from people with IBD [104] and/or use faecal calprotectin or endoscopic assessments [105]. In addition, the interviews were conducted by one of the investigators involved in recruitment of participants, which may have influenced the extent to which participants were willing to be critical. Lastly, the complete case analysis could have affected the external validity of the trial, as those who return the follow-up measures represent a non-random sample of the original group of participants [106]. However, due to the feasibility nature of the study [107] and the large amount of missing data (> 40%) for the 6 and 12 months’ follow-up [108, 109], the use of imputation methods could not be justified.

## Conclusions

Despite its limitations, this feasibility study adds useful and applicable knowledge to the management of IBD-fatigue. This is the first intervention to test the applicability of CBT treatment models for fatigue with proven effectiveness in other long-term conditions to people with IBD. The preliminary findings indicating greater long-term changes in severity and impact of fatigue in intervention Group 1 compared to control Group 2 indicate the need for further exploration of the use of CBT to improve IBD-fatigue. An adequately powered RCT is needed to investigate effectiveness, maintenance of treatment gains and the cost-effectiveness of the therapy. Incorporating changes to the protocol and an online intervention may ultimately be an effective way to overcome the barriers to implementation identified by HCPs and test the generalisability of the intervention to IBD-clinical practice.

## Supplementary information

**Additional file 1: Table S1.** Means, standard deviations, change scores and effect sizes of participants who completed baseline and 6-months follow-up primary and secondary outcome measures. **Table S2.** Means, standard deviations, change scores and effect sizes of participants who completed baseline and 12-months follow-up primary and secondary outcome.

## Data Availability

The datasets generated or analysed during the current study are not publicly available (individual privacy of the participants could be compromised) but are available from the corresponding author on reasonable request.

## Abbreviations

CBT: Cognitive-behavioural therapy
CCUK: Crohn’s and Colitis UK
CD: Crohn’s disease
CONSORT: Consolidated Standards of Reporting Trials
CRP: C-reactive Protein
GAD: Generalised anxiety disorder
HCP: Healthcare professional
IBD: Inflammatory bowel disease
MRC: Medical research council
MS: Multiple sclerosis
NHS: National Health Service
PPI: Patient and public involvement
QOL: Quality of life
RCT: Randomised controlled trial
RT: Relaxation therapy
SFT: Solution-focused therapy
UC: Ulcerative colitis
UK: United Kingdom
