# Supplementary material for: Cognitive-behavioural therapy for the management of inflammatory bowel disease-fatigue: a feasibility randomised controlled trial
Source: Pilot Feasibility Stud. 2019 Dec 10;5:145. doi: 10.1186/s40814-019-0538-y (PMC6905023; doi:10.1186/s40814-019-0538-y)
Supplement: Supplementary file 1 — Additional file 1: Table S1. Means, standard deviations, change scores and effect sizes of participants who completed baseline and 6-months follow-up primary and secondary outcome measures. Table S2. Means, standard deviations, change scores and effect sizes of participants who completed baseline and 12-months follow-up primary and secondary outcome. [file 40814_2019_538_MOESM1_ESM.docx]

**Online supplementary Table 1: Means, standard deviations, change scores and effect sizes of participants who completed baseline and 6-months follow-up primary and secondary outcome measures.**

| **Outcome** | **Group** | **Baseline** | |  | **6-months follow-up** | | | **Change Scores** | | | **Between groups effect size (CI)** |
| --- | --- | --- | --- | --- | --- | --- | --- | --- | --- | --- | --- |
|  |  | **Mean** | **SD** |  | **Mean** | **SD** | **N** | **Mean** | **SD** | **MD (CI)** |  |
| IBDF-1 | Group 1 | 12.00 | 2.83 |  | 7.60 | 4.04 | 5 | -4.40 | 2.41 | -2.90 (-7.39, - 6.88) | 0.83 (-0.27, 1.93) |
|  | Group 2 | 9.62 | 4.31 |  | 8.12 | 4.15 | 8 | -1.50 | 4.11 |  |  |
| IBDF-2 | Group 1 | 55.25 | 26.21 |  | 30.85 | 24.61 | 8 | -24.29 | 15.65 | -15.59 (-31.55, -39.99) | 1.30 (0.14, 2.47) |
|  | Group 2 | 33.43 | 22.21 |  | 24.63 | 27.14 | 9 | -8.69 | 8.38 |  |  |
| IBDQ | Group 1 | 87.00 | 15.54 |  | 91.83 | 18.70 | 6 | 4.83 | 11.90 | -2.91 (-15.18, 10.50) | 0.23 (-0.83, 1.29) |
|  | Group 2 | 93.75 | 10.11 |  | 100.87 | 10.85 | 8 | 7.1 | 7.99 |  |  |

*Key:* **CI-** Confidence Interval; **IBD-F;** Inflammatory Bowel Disease-Fatigue; **IBDQ;** Inflammatory Bowel Disease Questionnaire **MD-** mean difference; **SD-** Standard Deviation

**Online supplementary Table 2: Means, standard deviations, change scores and effect sizes of participants who completed baseline and 12-months follow-up primary and secondary outcome**

| **Outcome** | **Group** | **Baseline** | |  | **12-months follow-up** | | | **Change Scores** | | |  |
| --- | --- | --- | --- | --- | --- | --- | --- | --- | --- | --- | --- |
|  |  | **Mean** | **SD** |  | **Mean** | **SD** | **N** | **Mean** | **SD** | **MD (CI)** | **Between group effect sizes (CI)** |
| IBDF-1 | Group 1 | 12.00 | 2.58 |  | 8.43 | 2.51 | 7 | -3.57 | 1.72 | -1.77 (-4.62, 1.10) | 0.91 (-0.30, 2.11) |
|  | Group 2 | 9.80 | 2.77 |  | 8.00 | 2.65 | 5 | -1.80 | 2.28 |  |  |
| IBDF-2 | Group 1 | 47.60 | 23.49 |  | 32.75 | 19.80 | 5 | -14.85 | 20.14 | -14.53 (-39.55, 10.49) | 0.87 (-0.22, 2.07) |
|  | Group 2 | 22.50 | 8.54 |  | 22.18 | 14.33 | 4 | -0.32 | 9.58 |  |  |
| IBDQ | Group 1 | 87.14 | 14.47 |  | 97.57 | 8.88 | 7 | 10.43 | 10.29 | 8.43 (-1.74, 18.60) | 0.98 (-2.19, 0.23) |
|  | Group 2 | 98.00 | 4.74 |  | 100.00 | 4.30 | 5 | 2.00 | 5.10 |  |  |
